# Takotsubo Cardiomyopathy Caused by Carbon Dioxide Intoxication

**DOI:** 10.7759/cureus.14179

**Published:** 2021-03-29

**Authors:** Haruna Inoue, Takeshi Nishimura, Tsuyoshi Nojima, Hiromichi Naito

**Affiliations:** 1 Cardiology, Kobe Red Cross Hospital, Kobe, JPN; 2 Department of Emergency and Critical Care Medicine, Hyogo Emergency Medicine, Kobe, JPN; 3 Department of Emergency, Critical Care and Disaster Medicine, Okayama University Graduate School of Medicine, Dentistry and Pharmaceutical Sciences, Okayama, JPN

**Keywords:** drug intoxication, total carbon dioxide, takutsubo cardiomyopathy

## Abstract

A 49-year-old man transferred to our hospital for dyspnea that developed while transporting significant loads of dry ice, which may have caused potential carbon dioxide intoxication. On admission, he presented hyperventilation and disorientation. Transthoracic echocardiography showed the reduced motion of the anterior wall of the left ventricle with decreased left ventricular ejection fraction. The patient underwent coronary angiography, which did not show apparent coronary arterial stenosis. The electrocardiogram revealed T-wave change and echocardiography results showed the subsided changes on the third hospital day. He was discharged without any symptoms on the fourth hospital day. Our case demonstrates the potential association between carbon dioxide intoxication and Takotsubo cardiomyopathy. Our experience may inform emergency physicians in formulating diagnostic/therapeutic approaches for similar patients experiencing cardiac failure following carbon dioxide intoxication.

## Introduction

Dry ice has been identified as a cause of accidental carbon dioxide (CO2) intoxication, as it generates a large amount of CO2 by sublimation. The risk of acute poisoning due to inhalation of CO2 gas is less common, though a number of cases of suicide, murder, and even mass casualties caused by high concentrations of CO2 exposure have been reported [1]. CO2 acts as a toxicant, causing asphyxiation by hypoxia. CO2 intoxication influences the respiratory system, neurological function, and even the cardiovascular system. This is because increases in CO2 alter its buffering capacity, thereby changing cellular pH, which leads the to the breakdown of the normal buffering system and acidosis as well as an increase in the potassium concentration [2]. CO2 inhalation has been reported to be associated with catecholamine increase [3]. 

Although ischemic changes on electrocardiogram (ECG), including T-wave inversion and ST-segment changes, have been described in the previous literature [4], the direct cardiotoxicity of CO2 has not been elucidated. Herein, we report a rare case of Takotsubo cardiomyopathy presumably caused by CO2 intoxication. Although various factors, including the excess release of catecholamine, are known to be involved in the development of Takotsubo cardiomyopathy, the relationship between Takotsubo cardiomyopathy and CO2 intoxication has never been reported. Our report may aid emergency physicians in developing diagnostic and therapeutic approaches for similar cases of cardiac failure following CO2 intoxication.

## Case presentation

A 49-year-old man was admitted to our hospital for dyspnea. He had no past or present medical history or allergies. He had called an ambulance because his asphyxia had gradually worsened while driving with cardboard boxes containing dry ice. Emergency medical technicians arrived at the scene and found the patient inside the car with all doors closed. There were no smoke or odors inside the car to suspect intoxication. This patient presented with hyperventilation and was disoriented. His vital signs at the scene were as follows: heart rate of 102 beats per minute (bpm), blood pressure (BP) of 95/75mmHg, respiratory rate of 40 breaths per minute with hyperventilation, and Glasgow Coma Scale (GCS) score of E1V2M4. At prehospital, oxygen administration was started.

On arrival at the hospital, his airway was patent without respiratory distress. The patient presented with dizziness and headache. His vital signs on hospital arrival were as follows: heart rate 100 bpm, BP100/79 mmHg, oxygen saturation 98% with 6L per minute oxygen, respiratory rate 16 breaths per minute, and GCS score E3V5M6. His arterial blood gas data were as follows; pH 7.415, PCO2 39.3mmHg, PO2 133mmHg, HCO3 24.7mmol/L, anion gap 11.4mmol/L, and lactate 19.8mg/L. The patient presented with pale skin, general fatigue, and chest discomfort. According to an interview with his colleague, several of his colleagues had experienced dyspnea as well while transporting dry ice in their loading cars. Although his blood CO2 levels had already returned to a normal level, we made the clinical diagnosis that this patient’s symptoms were likely driven by CO2 intoxication caused by dry ice. 

**Figure 1:**
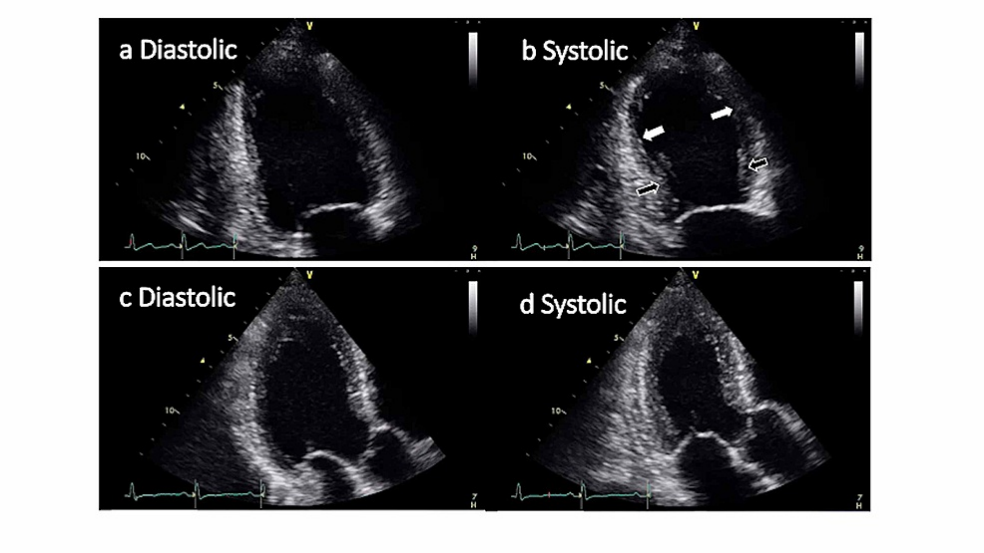
Transthoracic echocardiography a-b: Transthoracic echocardiography (TTE) on admission showed left ventricular desynchrony with decreased ejection fraction. The base of the left ventricle contracture was hyperkinetic (black arrowhead), while the rest of the left ventricle was akinetic or dyskinetic (white arrowhead). c-d: TTE on the third hospital day revealed that left ventricular dysfunction had recovered completely.

**Figure 2:**
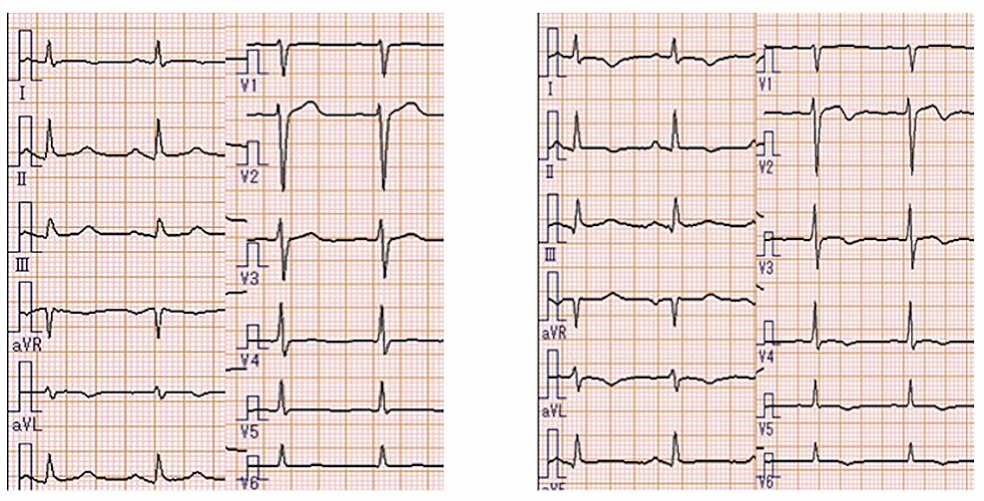
Electrocardiogram (Left) Electrocardiogram on admission showed normal findings without S-T change or T-wave inversion. (Right) ECG on third hospital day showed T-wave inversion in V2 - V6.

**Figure 3:**
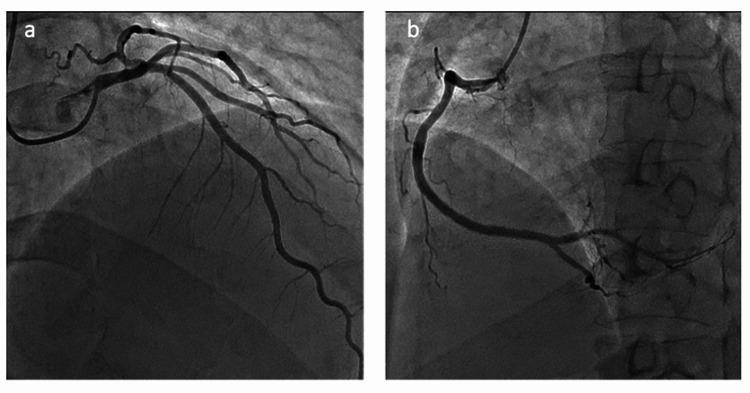
Coronary angiography Coronary angiography demonstrated no significant coronary obstructive stenosis (a. left coronary artery, b. right coronary artery).

No murmurs were noted, and his respiratory auscultation was clear. The radiograph indicated bilateral lung congestion. Transthoracic echocardiography (TTE) revealed left ventricular dysfunction, with an estimated left ventricular ejection fraction (EF) of 35% (Figures 1a-1b). Desynchrony between the base and apex of the left ventricular wall was revealed. The base of the left ventricle contracture was hyperkinetic, but the rest of the left ventricle was akinetic or dyskinetic. ECG on arrival showed normal sinus rhythm, 96 bpm, without ST change (Figure 2, left). Cardiac enzyme levels from laboratory tests performed on admission showed the following: troponin-T 0.01 ng/dl, creatinine kinase (CK) 97 IU/L, creatine kinase myocardial band (CK-MB) 2 ng/dL. On the second hospital day, CK and CK-MB increased respectively (CK 286 IU/L, CK-MB 12 ng/dL). On the third hospital day, TTE revealed that his left ventricular wall motion had returned to normal, with an estimated left ventricular EF of 60% (Figures 1c-1d), and the wall motion desynchrony had disappeared completely. ECG showed T-wave inversion in V2-V6 (Figure 2, right). Coronary angiography showed no significant coronary arterial stenosis (Figure 3). Based on the clinical manifestation and episodes, the patient was diagnosed with possible Takotsubo cardiomyopathy, presumably caused by CO2 intoxication. The patient was discharged on the fourth day of hospitalization without any complications.

## Discussion

The present case of Takotsubo cardiomyopathy was likely to have been induced by CO2 intoxication. We do not have clear evidence of direct cardiotoxicity by inhaled CO2; however, we assume that sublimation of dry ice generated CO2, which caused severe anxiety due to respiratory stimulation and disorientation, leading to catecholamine surge and development of Takotsubo cardiomyopathy. Immediate improvement of the symptoms after administration of oxygen therapies may support our diagnosis. Environmental factors like the confined space with the dry ice in the loading car with all doors closed and his colleagues’ similar episodes during transport may also reasonably support our diagnosis. 

CO2 is a dangerous poison that causes convulsions, coma, and death when the concentration exceeds 10% in the air [2]. The systemic effects of sympathetic stimulation have been reported, such as cardiac arrhythmia, increasing heart rate, pulmonary vascular resistance, and pulmonary artery pressure [1]. Furthermore, higher CO2 concentration causes the development of hyperventilation and respiratory acidosis, which increase parasympathetic nervous activity and interfere with the acetylcholine hydrolysis by acetylcholinesterase, resulting in circulatory and respiratory depression [5]. 

The diagnosis of CO2 intoxication is challenging and mostly made based on-scene investigation and circumstances as well as measuring the concentration of CO2, since there are no unique characteristic clinical manifestations of CO2 intoxication. Blood tests will reveal high CO2 levels, but these tests are not always available before hospital arrival and the concentration of partial CO2 in the blood rapidly decreases with oxygen therapies [6]. A previous study showed a wide range of blood CO2 concentrations (41.8-64.6 mmHg) among symptomatic subjects since patients present asphyxiation quickly substituted by hyperventilation [7]. In our patient, the partial CO2 pressure at the time of hospital arrival was almost normal. 

Dry ice is a common cause of CO2 intoxication and is widely used in daily life as a cooling agent in a solidified form that sublimates at -78.5°C [8]. The clinician should be aware of the possibility of CO2 intoxication when encountering coma patients rescued from confined spaces. 

Triggers of Takotsubo cardiomyopathy, including physical and emotional factors, are well described in the literature. Takotsubo cardiomyopathy occurs mainly in postmenopausal women [9]. Males develop the condition less frequently than females, and it is often associated with physical stress [10]. Results from a meta-analysis of patients with Takotsubo syndrome were that 39% had emotional stressors, 35% had physical stressors, and 17% had no stressors [4]. Several cases of Takotsubo cardiomyopathy triggered by intoxication have been reported, and almost all cases were associated with intentional drug intoxication per oral consumption such as suicide attempts [11]. Currently, there are no reports of Takotsubo cardiomyopathy triggered by CO2 intoxication to the best of our knowledge. 

Takotsubo cardiomyopathy, which is named for the appearance of the left ventricular dysfunction during systole, is related to cardiovascular functional collapse associated with catecholamine increase [9]. This syndrome mimics acute coronary syndrome, with the common symptoms of dyspnea, chest pain, elevated cardiac enzymes, and ST-segment elevation or T-wave inversion on ECG, but no obstructive coronary disease or angiographic evidence of acute plaque rupture [12]. The pathophysiological mechanism of Takotsubo cardiomyopathy remains unknown. However, catecholamine concentration in the blood is two to three times higher than usual, accompanied by, in particular, a marked increase in adrenaline [13]. Adrenaline stimulates β2-adrenergic receptors, which are abundant in the apex and have been shown to shift to inhibitory signals by a signal switching mechanism. It leads to apical contractile dysfunction. In contrast, there are many sympathetic nerve endings at the base of the heart, resulting in cardiac base hypercontraction due to sympathetic nerve stimulation. Thus, the imbalance between the apex and cardiac base wall motion is typical of Takotsubo cardiomyopathy [14]. Interestingly, some ECG variations (changes in QT interval prolongation, P-wave morphology, ST-segment, T-wave, and non Q-wave myocardial infarction) recognized in Takotsubo cardiomyopathy cases have also been observed in CO2 intoxication cases [15]. Although changes in CO2 intoxication on ECG have been reported, the morphology of the heart is unknown. As far as we know, no reports about the heart’s movement visualized by echocardiography during CO2 intoxication have been published. 

Based on patients’ symptoms, examinations, and surroundings, we hypothesize that CO2 intoxication induced by dry ice sublimation might cause stress and increased catecholamine levels, which selectively act on the part of the myocardium and cause impaired contraction following complex Takotsubo-like wall motion dysfunction. 

## Conclusions

We experienced a rare case of Takotsubo cardiomyopathy which was likely caused by CO2 intoxication. This mechanism is complex, but emergency clinicians and cardiologists should be aware of the unique etiology of this condition and give appropriate therapy when treating CO2 intoxication patients.
